# siRNA-Mediated Simultaneous Regulation of the Cellular Innate Immune Response and Human Respiratory Syncytial Virus Replication

**DOI:** 10.3390/biom9050165

**Published:** 2019-04-28

**Authors:** María Martín-Vicente, Salvador Resino, Isidoro Martínez

**Affiliations:** Unidad de Infección Viral e Inmunidad, Centro Nacional de Microbiología, Instituto de Salud Carlos III, Majadahonda, 28220 Madrid, Spain; maria.martinv@externos.isciii.es

**Keywords:** respiratory syncytial virus, siRNA, innate immune response, treatment

## Abstract

Human respiratory syncytial virus (HRSV) infection is a common cause of severe lower respiratory tract diseases such as bronchiolitis and pneumonia. Both virus replication and the associated inflammatory immune response are believed to be behind these pathologies. So far, no vaccine or effective treatment is available for this viral infection. With the aim of finding new strategies to counteract HRSV replication and modulate the immune response, specific small interfering RNAs (siRNAs) were generated targeting the mRNA coding for the viral fusion (F) protein or nucleoprotein (N), or for two proteins involved in intracellular immune signaling, which are named tripartite motif-containing protein 25 (TRIM25) and retinoic acid-inducible gene-I (RIG-I). Furthermore, two additional bispecific siRNAs were designed that silenced F and TRIM25 (TRIM25/HRSV-F) or N and RIG-I (RIG-I/HRSV-N) simultaneously. All siRNAs targeting N or F, but not those silencing TRIM25 or RIG-I alone, significantly reduced viral titers. However, while siRNAs targeting F inhibited only the expression of the F mRNA and protein, the siRNAs targeting N led to a general inhibition of viral mRNA and protein expression. The N-targeting siRNAs also induced a drastic decrease in the expression of genes of the innate immune response. These results show that both virus replication and the early innate immune response can be regulated by targeting distinct viral products with siRNAs, which may be related to the different role of each protein in the life cycle of the virus.

## 1. Introduction

Human respiratory syncytial virus (HRSV) is an enveloped, single-stranded, negative-sense RNA virus belonging to the *Orthopneumovirus* genus within the *Pneumoviridae* family, whose genome contains 10 genes encoding 11 proteins [1,2]. Three of them (F, G, and SH) are glycoproteins inserted in the viral envelope. F and G are involved in virus attachment and entry into target cells, while the role of SH is less clear. Five proteins (N, P, L, M2-1, and M2-2) are involved in viral RNA replication and transcription. An additional structural protein (M) forms a protein layer under the viral envelope and participates in virus maturation. Lastly, two non-structural proteins (NS1 and NS2) have been implicated in counteracting the host immune response to HRSV infection [3,4].

Human respiratory syncytial virus is a recognized important pathogen that causes serious low tract respiratory infections in infants, the elderly, and immunocompromised people [5,6], and it is also related to the exacerbation of asthma and chronic obstructive disease [7,8]. It is estimated that HRSV produces more than 33 million low respiratory infections in children under five years old each year. Approximately 10% of them require hospitalization and about 60,000 die, mostly in developing countries [9,10].

The immune response against HRSV begins in the infected respiratory epithelial cells, where viral RNA is recognized by pattern recognition receptors (PRRs), mainly retinoic acid-inducible gene-I (RIG-I)-like and Toll-like receptors, which leads to the induction of type I and III interferons, inflammatory cytokines, and antiviral genes [11]. The RIG-I is a cytosolic PRR that recognizes pathogen-specific uncapped RNAs, which trigger the induction of innate immune genes [12]. Retinoic acid-inducible gene-I, which is encoded by the *DExD/H-box helicase 58* (*DDX58*) gene, is essential for the development of the early anti-HRSV response [13], and its activity is tightly regulated to ensure proper termination of immune signaling after virus clearance. This regulation is mainly mediated by processes of ubiquitination and deubiquitination in which the E3 ubiquitin ligase tripartite motif-containing protein 25 (TRIM25) has a prominent role [14]. The TRIM25-mediated activation of RIG-I is essential for the initiation of the intracellular antiviral response, although TRIM25 may also negatively regulate RIG-I through a different mechanism [15].

Despite its health impact, there is no available vaccine or effective treatment against this virus, although intensive efforts are being made in both fields [16,17]. In addition to this, the strong inflammation associated with serious HRSV infections of the lower respiratory tract does not respond to conventional anti-inflammatory treatments [18]. As a result of all these issues in preventing and treating HRSV infections, intensive research is being done looking for alternative approaches, such as the development of antivirals or treatment with immunomodulatory molecules aiming to moderate the viral-induced pathology.

Recently, synthetic small interfering RNAs (siRNAs) have emerged as promising molecules to treat several pathologies, including cancer, inflammatory disorders, and metabolic and infectious diseases [19,20]. Pioneering studies showing the efficacy of siRNAs against viruses included HRSV [21], human immunodeficiency virus-1 (HIV-1) [22], and the influenza virus [23,24]. Afterward, several studies have reported the inhibition of HRSV by siRNA-mediated silencing of a number of HRSV genes, including *NS1*, *N*, *P*, *F,* and *M2-2* [25,26,27,28,29,30,31,32,33,34]. Some of them have been tested in vivo in the mouse model and found to restrict HRSV replication [25,27,32]. These siRNAs were administered intranasally and they were effective even when delivered free of transfection reagents and in prophylaxis or treatment models [25,27,32]. These results encouraged further investigations, and an siRNA (ALN-RSV01) targeting HRSV N was shown to significantly reduce the number of virus infections in healthy adults in a Phase 2 study [28]. The ALN-RSV01 was tested in lung transplant patients in a Phase 2 clinical trial [29,31]. However, although it decreased the rate of new or progressive bronchiolitis obliterans syndrome, there was no significant effect on viral parameters or symptom scores [31].

With the aim of identifying new molecules to treat HRSV infection, in the present study, we have designed two bispecific siRNAs that simultaneously target the HRSV F and TRIM25 mRNAs or the HRSV N and RIG-I mRNAs. The effect of these bispecific siRNAs was compared to monospecific siRNAs silencing F, N, TRIM25, or RIG-I alone. Both bispecific siRNAs and monospecific siRNAs targeting viral genes reduced virus growth and proinflammatory cytokine production. Notably, while siRNAs targeting N and F reduced virus growth to a similar extent, they differed markedly in the inhibition of viral RNA and protein accumulation as well as in the attenuation of the inflammatory/antiviral response.

## 2. Materials and Methods

### 2.1. Cells and Virus

Human lung carcinoma cells (A549) and human carcinoma HeLa-derived cells (HEp-2) were maintained in Dulbecco’s modified Eagle’s medium (DMEM, HyClone, Logan, Utah, USA) supplemented with 10% Fetal Bovine Serum (FBS, Biological Industries, Beit HaEmek, Israel), 4 mM l-Glutamine (HyClone), 100 U/mL penicillin (Lonza, Verviers, Belgium), and 100 µg/mL streptomycin (HyClone) (DMEM10). Cells were cultured at 37 °C in a 5% CO_2_ atmosphere. HEp-2 cells were infected with the HRSV Long strain and the virus was purified from clarified culture supernatants by polyethylene glycol precipitation and centrifugation in a discontinuous sucrose gradient, as previously described [35,36]. The purified virus was aliquoted and stored in liquid nitrogen as a stock for subsequent infections.

### 2.2. Viral Infections and Plaque Assays

A549 were infected with HRSV at a multiplicity of infection (MOI) of 3 or 0.1 plaque-forming units (pfu) per cell in DMEM 2% FBS (DMEM2). Virus adsorption was allowed for 90 min at 37 °C. After this time, fresh DMEM2 was added. Culture supernatants and cell pellets were collected at different times post-infection. Virus titers were determined in the supernatants and total RNA and proteins were extracted from the cell pellets.

For HRSV titration, HEp-2 cell monolayers were incubated with serial dilutions of the infected cell supernatants for 90 min at 37 °C and then covered with 0.7% agarose (LM Sieve, Conda, Madrid, Spain) in DMEM2. Agarose was allowed to harden during 45 min at 4 °C, and then cells were transferred to a CO_2_ incubator at 37 °C for five days. After this, cells were fixed with 4% formaldehyde in PBS for 45 min, which was followed by methanol permeabilization for 10 min. Virus plaques were visualized by one hour incubation with a mixture of monoclonal antibodies against HRSV [35], followed by one hour incubation with an anti-mouse IgG horseradish-peroxidase-linked whole antibody (Abcam, Cambridge, UK), and 3-amino-9-ethylcarbazole (AEC, Alfa Aesar, Ward Hill, MA, USA).

### 2.3. Quantitative Real Time-PCR

Total RNA from treated or controlled cells was purified with the ReliaPrep RNA Cell Miniprep System (Promega, Madison, WI, USA) and was reverse transcribed with the High-Capacity cDNA Reverse Transcription Kit (Applied Biosystems, Foster City, CA, USA) following the manufacturer’s instructions. Gene expression was quantitated in triplicate by RT-PCR (qRT-PCR) with a Step One instrument (Applied Biosystems, Foster City, CA, USA) following the manufacturer’s instructions.

TaqMan MGB probes (FAM dye-Labeled, Applied Biosystems) were used for the following cellular genes: *tripartite motif-containing 25* (*TRIM25*) (Hs01116121_m1), *DExD/H-Box Helicase 58* (*DDX58*) (RIG-I, Hs00204833_m1), *actin-β* (*ACTB*) (Hs99999903_m1), *interleukin 6* (*IL6*) (Hs00985639_m1), *interferon-stimulated gene 15* (*ISG15*) (Hs00192713_m1), *interferon-β1* (*IFNB1*) (Hs01077958_s1), *tumor necrosis factor* (*TNF*) (Hs00174128_m1), and *chemokine C-C motif ligand 5* (*CCL5*) (Hs00982282_m1). Gene expression was normalized to *ACTB* expression and relative quantifications were made by the comparative CT (ΔΔC_T_) method.

Expression of HRSV genes was performed, according to the manufacturer’s instructions, using a SYBR-Green reaction mix (Power-Up SYBR-Green Master MIX, Applied Biosystems) and the following primers: *N* (forward: 5′-CATGATTCTCCTGATTGTGGGATGA-3′, reverse: 5′-TCACGGCTGTAAGACCAGATCTAT-3′); *F* (forward: 5′-ACCAGCAAAGTGTTAGACCTCAA-3′, reverse: 5′-TCCCTGGTAATCTCTAGTAGTCTG-3′); *NS1* (forward: 5′-GCTTTGGCTAAGGCAGT GAT-3′, reverse: 5′-CCATTAGGTTGAGAGCAATGTG-3′); *L* (forward: 5′-TGGCAGTTACAGAGGTTTTG-3′, reverse: 5′-GCCCGTGAGGATATGTAGGTT-3′); *G* (forward: 5′-CCTCAGCTTGGAATCAGCTT-3′, reverse: 5′-GTGGTTTGTTTTGGCGTTGTTT-3′); *M* (forward: 5′-ACCCAAGGGACCTTCACT-3′, reverse: 5′-GTGTGGGGTTGAGTGTCTTC-3′); and *P* (forward: 5′-GCTAGGGATGGTATAAGAGATGCC-3′, reverse: 5′-CTCTGATGTTGGATTGAGAGACACT-3′). Expression of *ACTB* (*β-actin*) (forward: 5′-CACCAACTGGGACGACAT-3′, reverse: 5′ACAGCCTGGATAGCAACG3′) was used for normalization. Relative quantifications were made by the comparative CT (ΔΔC_T_) method.

### 2.4. Western Blot

Protein levels were analyzed by a Western blot. Cell pellets were lysed in 1% Triton–1% Sodium Deoxycholate–0.1% Sodium Dodecyl Sulfate (SDS) buffer with protease inhibitors (Complete Mini, Roche, Indianapolis, IN, USA) and protein concentration was determined by the Bicinchoninic acid (BCA) protein assay (Pierce BCA protein assay, Thermo Scientific, Rockford, IL, USA). A total of 10 µg of each protein sample was separated in 10% SDS-Polyacrylamide gel electrophoresis gels and subsequently transferred to an immobilon-P membrane (Millipore, Burlinton, MA, USA). Primary antibodies for detection of the following proteins were used: actin-β (Ab8224-100, Abcam), HRSV matrix protein (29 M), HRSV G glycoprotein (021/1 G and 021/2 G), HRSV fusion protein (476–510), and HRSV nucleoprotein (79 N) [37,38]. Horseradish-peroxidase-linked anti-rabbit or anti-mouse Ig (Abcam) were used as secondary antibodies. Proteins were visualized by chemiluminescence using the SuperSignal West Pico Chemiluminescent Substrate (Thermo Fisher, Rockford, IL, USA) in a ChemiDoc MP Imaging System (Bio-Rad, Hercules, CA, USA).

### 2.5. Small Interferring RNA Design

Small interfering RNAs against *DDX58* (*RIG-I*) (ID # s223614) and *TRIM25* (ID # s15206 and ID # s15204) were purchased from Ambion (Themo Fisher) (Rockford, IL, USA). The latter siRNA (ID # s15204) was found to silence the HRSV fusion protein in addition to *TRIM25,* and it is referred to as TRIM25/HRSV-F siRNA (Table 1). Following previously described guidelines [39,40], an siRNA silencing the F glycoprotein alone (HRSV-F) (Table 1) was designed and the sequence was sent to Ambion for siRNA synthesis. The siRNA targeting the HRSV nucleoprotein (HRSV-N) was also synthesized by Ambion, and it is based on the sequence of the previously reported siRNA ALN-RSV01 [27] (Table 1).

**Figure 1:**
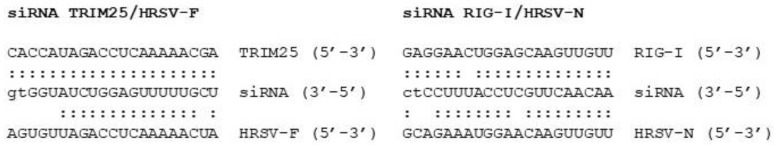
Nucleotide sequence of bispecific siRNAs against viral and cellular genes. The 21-nucleotide long sequence of the two bispecific siRNAs (antisense) silencing TRIM25 and HRSV-F (TRIM25/HRSV-F) or RIG-I and HRSV-N (RIG-I/HRSV-N) is shown. Dinucleotide overhangs are in lowercase. Canonical base pairing with TRIM25, RIG-I, HRSV-F, or HRSV-N mRNAs are represented by colons.

Lastly, a bispecific siRNA targeting the HRSV nucleoprotein and RIG-I (RIG-I/HRSV-N) was designed using LALIGN software [41]. Human respiratory syncytial virus genome (positive polarity) was aligned with the RIG-I mRNA. A region of 21 nucleotide residues with high identity between the two RNAs was selected and a bispecific siRNA was designed following previously described rules [39,40] and synthesized by Ambion (Figure 1, Table 1).

### 2.6. Small Interfering RNA Silencing

Forty-five thousand A549 cells were seeded in 24-well plates and transfected 24 h later with 6 pmol of control (negative control #2, Ambion, Thermo Fisher) or specific siRNAs and 1 µL of Lipofectamine RNAiMAx reagent (Invitrogen, Thermo Fisher) per well, following the manufacturer’s protocols. Twenty-four hours after transfection, cells were infected with HRSV at a MOI of 3. Additional experiments were done in which cells were first infected at MOI of 3 or 0.1 and then transfected 8 h later. Cell supernatants for viral titration and cell pellets for RNA and protein extraction were harvested at different hours post-infection, as indicated in the figure legends.

### 2.7. Cell Viability Assay

Viability of cells transfected with the different siRNAs was determined using an MTT [3-(4,5-dimethylthiazol-2-yl)-2,5-diphenyltetrazolium] assay. A549 cells (9 × 10^3^) were seeded onto 96-well plates. The following day, cells were transfected in quintuplicate with the different siRNAs at the same molar concentration as in 2.6. Ten µl of MTT (5 mg/mL, Calbiochem, San Diego, CA, USA) were added to each well at 0 and 72 h post-transfection, and cells were incubated for 4 h at 37 °C. After the incubation period, 120 µL per well of isopropanol with 0.04 N HCl were added and the plates were shaken for an additional hour. Absorbance (A) was measured at 570 nm and the survival rate was measured as SR (%) = (A treatment/A control) × 100%.

## 3. Results

### 3.1. Alignment of the Human Respiratory Syncytial Virus Genome with Innate Immune Genes and Selection of Bispecific Small Interfering RNAs

Since both virus replication and an inadequate inflammatory response seem to contribute to the severe pathology caused by HRSV, we wondered if it would be possible to design simple molecules to have an effect on the two processes at the same time. Specifically, we focused on designing single bispecific siRNAs able to simultaneously regulate the expression of viral and cellular innate immune genes. To do this, pairwise alignment was performed between the HRSV genome (positive polarity) and mRNAs from different genes involved in the regulation of intracellular immune pathways. These alignments revealed short segments of 12–14 nucleotides with an identical sequence between the virus and some cellular genes. These sequences may serve as targets for siRNAs modulating both virus production and the innate immune response. Based on these sequences, several siRNAs were designed to silence combinations of viral (*NS1*, *N*, *F*, *L*, trailer) and cellular genes (*TNF Alpha Induced Protein 3 (TNFAIP3/A20*), *Tax1 Binding Protein 1 (TAX1BP1*), *Cylindromatosis* (*CYLD*), *DExD/H-Box Helicase 58 (DDX58/RIG-I), TNF Receptor Associated Factor 3* (*TRAF3*). and *Tripartite Motif Containing 25 (TRIM25*)). Only two out of all the designed siRNAs were able to silence both viral and cellular genes simultaneously with one targeting *TRIM25* and *F* (TRIM25/HRSV-F) and the other one targeting *DDX58/RIG-I* and *N* (RIG-I/HRSV-N) (Figure 1 and Supplemental Figures S1, S2, and S3). TRIM25/HRSV-F is, in fact, a commercial siRNA designed by Ambion (ID # s15204) to silence *TRIM25* that we found also targets HRSV-*F*. TRIM25/HRSV-F matched 21 nucleotides out of 21 with TRIM25 mRNA and 15 nucleotides out of 21 with HRSV-F mRNA (Figure 1). RIG-I/HRSV-N matched 20 nucleotides out of 21 with RIG-I mRNA and 18 nucleotides out of 21 with HRSV-N mRNA (Figure 1). The DNA dinucleotide overhangs contributed to base pairing in most cases (Figure 1).

To compare the bispecific siRNAs, two additional monospecific siRNAs were designed and synthesized targeting *F* or *N*, and two others targeting *TRIM25* or *RIG-I* were purchased from Ambion (Table 1 and Supplemental Figures S2 and S3). The HRSV-N monospecific siRNA has an identical sequence to the previously described ALN-RSV01 [27]. All siRNAs were 21 nucleotides long, had a DNA dinucleotide overhang (Table 1), targeted sequences inside the coding region of the mRNAs (Supplemental Figures S2 and S3), and silenced the corresponding genes by more than 60% at 48 h post-transfection (Supplemental Figure S1a). TRIM25 and RIG-I downregulation was also measured at 24 h post-transfection (before infection) in the case of cells transfected with TRIM25, RIG-I, TRIM25/HRSV-F, or RIG-I/HRSV-N siRNAs. Even at this early post-transfection time, a clear inhibition of the expression of the corresponding genes was observed (Supplemental Figure S1b). None of the siRNAs tested were cytotoxic when transfected in A549 cells and compared to control cells transfected with no siRNA (Supplemental Figure S4).

### 3.2. Small Interfering RNAs Targeting F or N Inhibit Virus Growth

**Figure 2:**
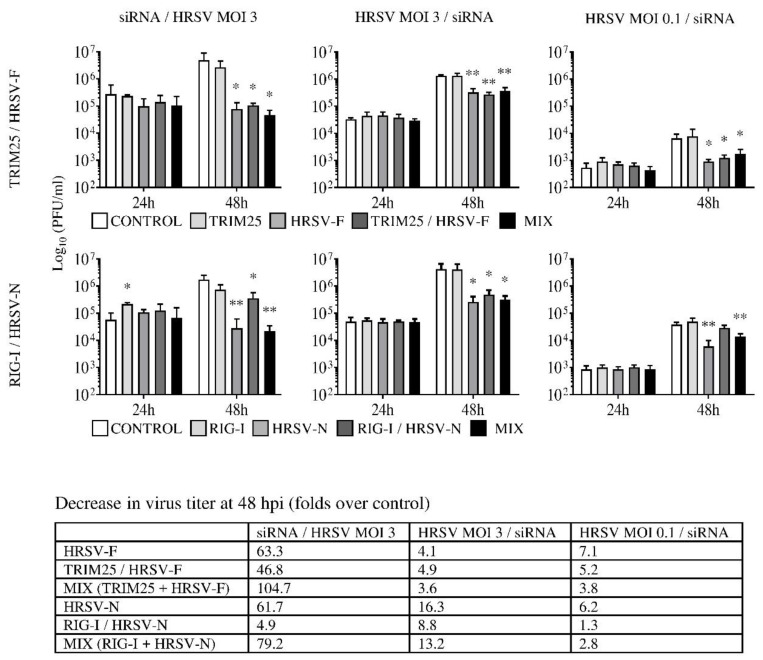
Human respiratory syncytial virus titers in the supernatant of infected cells transfected with the different siRNAs (upper panel: control, TRIM25, HRSV-F, TRIM25/HRSV-F or a combination of TRIM25 and HRSV-F (MIX); lower panel: control, RIG-I, HRSV-N, RIG-I/HRSV-N or a combination of RIG-I and HRSV-N (MIX)). Cells were transfected with the siRNAs before or after virus infection and the supernatant was collected at 24 and 48 h post infection (hpi). Small interfering RNA/HRSV multiplycity of infection (MOI) 3 (siRNA/HRSV MOI 3): infection was carried out at 24 h post-transfection at an MOI of 3. Human respiratory syncytial virus MOI 3/siRNA (HRSV MOI 3/siRNA): cells were infected at an MOI of 3 and then transfected eight hours later. Human respiratory syncytial virus MOI 0.1/siRNA (HRSV MOI 0.1/siRNA): infection was done at an MOI of 0.1 and transfection was carried out eight hours later. Data represent the mean and standard deviation from three independent experiments. Comparisons between groups were done by the *t*-test: *, *p* < 0.05 and **, *p* < 0.01. For 48 hpi, the decrease in the virus titer (fold over control) for each siRNA and condition is shown in the table below the graphics.

Cells were silenced with individual or mixed monospecific siRNAs, or with bispecific siRNAs in three distinct conditions of infection/transfection and virus titers were quantified by a plaque assay (Figure 2): (1) transfection of siRNAs and infection 24 h later at an MOI of 3. (2) Infection at an MOI of 3 and siRNA transfection 8 h later. (3) Infection at an MOI of 0.1 and siRNA transfection 8 h later. These three different conditions allow for the testing of three different treatment scenarios: (1) as prophylactic treatment before infection, (2) as therapeutic treatment in which nearly 100% of cells are infected, and (3) as inhibitors of virus growth and spread in conditions in which only about 10% of cells are infected.

Under all three conditions, when compared to cells transfected with a control siRNA, a significant decrease in virus titers was observed at 48 h post infection (hpi) with bispecific siRNAs (TRIM25/HRSV-F or RIG-I/HRSV-N), monospecific siRNAs targeting viral F (HRSV-F) or N (HRSV-N), or mixtures of siRNAs against viral F or N (TRIM25 + HRSV-F or RIG-I + HRSV-N) (Figure 2). No significant decrease in virus titers was observed with siRNAs targeting TRIM25 or RIG-I alone (Figure 2).

Reduction in virus titers were, however, about 10× greater when the siRNAs were applied before the infection than after the infection (Figure 2). Administration of the siRNAs before HRSV prevented the progression of the infection, since virus titers did not increase from 24 hpi to 48 hpi (Figure 2). By contrast, virus titers increased from 24 hpi to 48 hpi when siRNAs were transfected after HRSV infection (Figure 2). This increase was less evident in the case of siRNAs targeting F and infections at an MOI of 0.1 (Figure 2).

The siRNAs HRSV-F, TRIM-25/HRSV-F, and the combination of TRIM25 plus HRSV-F had a similar effect on virus production (Figure 2). Likewise, HRSV-N and the combination of RIG-I plus HRSV-N inhibited virus growth to the same extent, even though the bispecific RIG-I/HRSV-N was a little less effective, especially when administered prior to infection (Figure 2).

### 3.3. Silencing N, but Not F, Leads to a Generalized Inhibition of Human Respiratory Syncytial Virus RNA and Proteins

**Figure 3:**
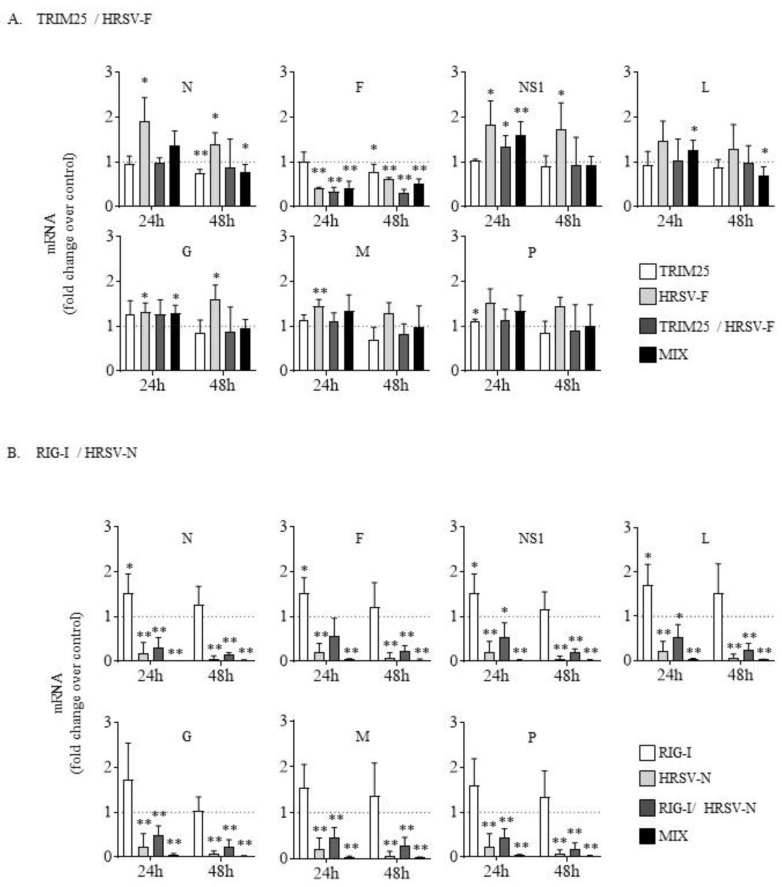
Relative levels of HRSV mRNAs (N, F, NS1, L, G, M, and P) in cells transfected with the different siRNAs. (**A**) Cells transfected with the siRNAs TRIM25, HRSV-F, TRIM25/HRSV-F or a combination of TRIM25 plus HRSV-F (MIX). (**B**) Cells transfected with the siRNAs RIG-I, HRSV-N, RIG-I/HRSV-N or a combination of RIG-I plus HRSV-N (MIX). Cells were transfected with the siRNAs and infected 24 h later at an MOI of 3. Levels of HRSV mRNAs were quantified by quantitative real time-PCR at 24 and 48 hpi and represented as fold over viral mRNAs expressed in cells transfected with a control siRNA. Data represent the mean and standard deviation from three independent experiments. Comparisons between groups were done by the *t*-test: *, *p* < 0.05 and **, *p* < 0.01.

**Figure 4:**
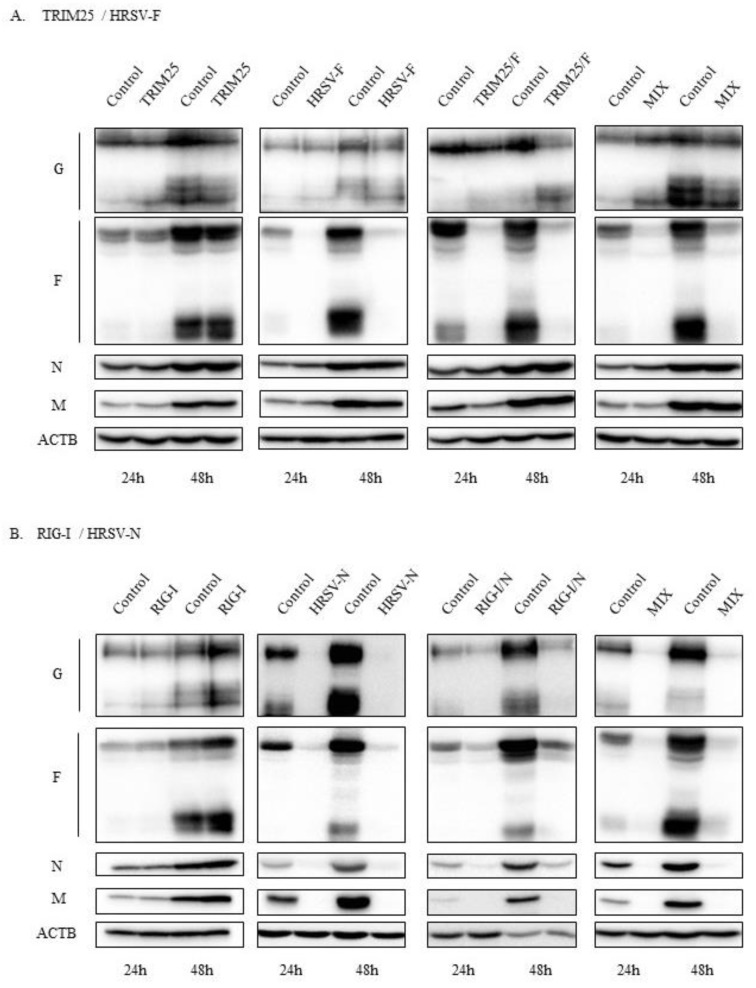
Levels of HRSV proteins (G, F, N, and M) in cells transfected with different siRNAs. (**A**) Cells transfected with the siRNAs TRIM25, HRSV-F, TRIM25/HRSV-F or a combination of TRIM25 plus HRSV-F (MIX). (**B**) Cells transfected with the siRNAs RIG-I, HRSV-N, RIG-I/HRSV-N or a combination of RIG-I plus HRSV-N (MIX). Cells were transfected with the siRNAs and infected 24 h later at an MOI of 3. Protein extracts were done at 24 and 48 hpi. The Western blot was utilized to analyze the cells using specific antibodies. The levels of β-actin (ACTB) are shown for comparison.

Since a greater inhibition in the viral titer was observed when siRNA transfection was performed before infection, we further investigated the levels of several viral mRNAs and proteins under this condition (Figure 3 and Figure 4).

While having a similar effect on viral titers, siRNAs targeting F or N differed substantially in their impact on viral mRNA and protein expression. The siRNAs targeting F (HRSV-F, TRIM25/HRSV-F and the combination of TRIM25 plus HRSV-F) significantly decreased the levels of the F mRNA, but not the other viral gene transcripts, both at 24 hpi and 48 hpi (Figure 3A). By contrast, siRNAs targeting N (HRSV-N, RIG-I/HRSV-N and the combination of RIG-I plus HRSV-N) drastically reduced the mRNA levels of all viral proteins tested (N, F, NS1, L, G, M, and P), both at 24 hpi and 48 hpi (Figure 3B). Again, the bispecific siRNA RIG-I/HRSV-N was a little less effective than HRSV-N or the combination of RIG-I plus HRSV-N at reducing mRNA (Figure 3B). As expected, siRNAs that targeted TRIM25 or RIG-I alone did not reduce viral mRNA levels (Figure 3).

Essentially, identical results were observed when the accumulation of viral proteins was analyzed by Western blotting (Figure 4). Silencing with HRSV-F, TRIM-25/HRSV-F, and the combination of TRIM25 plus HRSV-F resulted in a large decrease in F protein accumulation, but they had no effect on the levels of the other viral proteins (Figure 4A). By contrast, HRSV-N, RIG-I/HRSV-N, and the combination of RIG-I plus HRSV-N siRNAs silenced the expression of all viral proteins studied (Figure 4B). Once more, the effect of the bispecific RIG-I/HRSV-N, although substantial, was slightly less than that of HRSV-N or the combination siRNAs (Figure 4B). As anticipated, neither TRIM25 nor RIG-I siRNAs reduced the accumulation of viral proteins (Figure 4).

### 3.4. Targeting Human Respiratory Syncytial Virus Nucleoprotein with Small Interfering RNAs Drastically Reduces the Cellular Innate Immune Response While Human Respiratory Syncytial Virus Fusion Protein Silencing Has Only a Moderate Effect

The intracellular innate immune response induced by HRSV was analyzed under the three different conditions of siRNA transfection by measuring mRNAs levels of interleukin 6 (IL6), ISG15 ubiquitin-like modifier (ISG15), tumor necrosis factor-alpha (TNF-α), C-C motif chemokine ligand 5 (CCL5), and interferon beta 1 (IFN-β).

**Figure 5:**
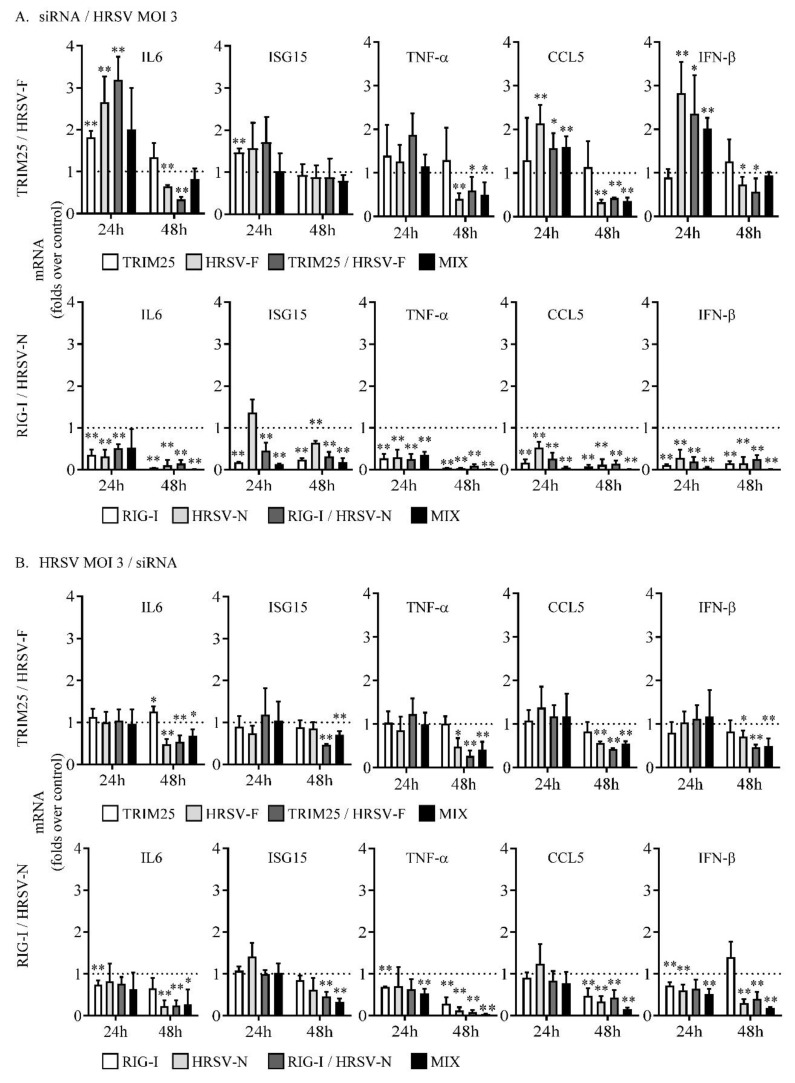

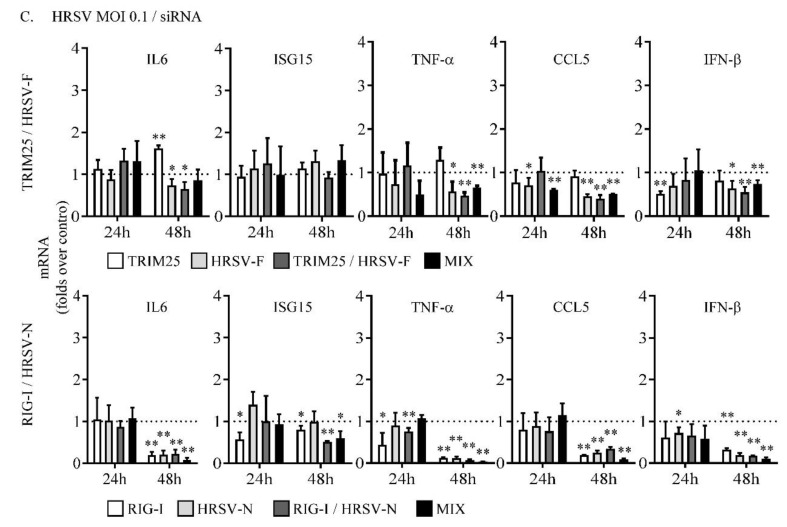
Relative levels of mRNAs from genes involved in the antiviral immune response in cells transfected with the different siRNAs. (**A**) Cells were transfected with the indicated siRNAs and infected 24 h later at a MOI of 3. (**B**) Cells were infected at a MOI of 3 and transfected 8 h later with the indicated siRNAs. (**C**) Cells were infected at a MOI of 0.1 and transfected 8 h later with the indicated siRNAs. Transfection/infection were done as in Figure 2. Levels of mRNAs were quantified by qRT-PCR at 24 and 48 hpi and represented as fold over mRNAs expressed in cells transfected with a control siRNA. Data represent the mean and standard deviation from three independent experiments. Comparisons between groups were done by using the *t*-test: *, *p* < 0.05 and **, *p* < 0.01.

In general, targeting HRSV F with HRSV-F siRNA, TRIM25/HRSV-F siRNA, or the combination of HRSV-F plus TRIM25 siRNAs moderately reduced the expression of IL6, ISG15, TNF-α, CCL5, and IFN-β at 48 hpi, but not at 24 hpi (Figure 5). However, ISG15 levels were not significantly reduced in the cases in which the siRNAs were administered before infection (siRNA/HRSV MOI 3, Figure 5A) or when infection at MOI 0.1 was carried out before siRNA transfection (HRSV MOI 0.1/siRNA, Figure 5C). When the siRNAs HRSV-F, TRIM25/HRSV-F, or the combination of TRIM25 plus HRSV-F were transfected before infection, the levels of IL6, CCL5, and IFN-β mRNAs increased significantly at 24 hpi (Figure 5A). Tripartite motif-containing protein 25 only silencing did not result in a decrease in the innate immune response in any case (Figure 5).

By contrast, when the expression of HRSV N was reduced by the siRNAs HRSV-N, RIG-I/HRSV-N, and the combination of RIG-I plus HRSV-N, a strong decrease in the innate immune response was observed at 48 hpi (Figure 5). In addition, this response was also inhibited at 24 hpi when siRNAs were administered before the infection (Figure 5A), and, in some cases, when delivered after infection (Figure 5B,C). Unlike what happened with the monospecific siRNA targeting TRIM25, RIG-I siRNA alone was able to considerably reduce the innate immune response (Figure 5). This discrepancy between the effects of TRIM25 and RIG-I siRNAs may be related to the fact that TRIM25 has a dual role in RIG-I regulation, since it also negatively regulates RIG-I through an indirect mechanism [15]. In addition, other E3 ubiquitin ligases may compensate for the TRIM25 downregulation [12].

## 4. Discussion

While great efforts are being made to find prophylactic and therapeutic treatments for HRSV and to understand the role of the immune response in the development of the HRSV-induced pathology, no approved vaccine or effective therapeutic treatment is currently available for this virus. In the search for novel treatments for HRSV, we have explored the idea of inhibiting both the virus replication and the innate immune response by designing bispecific siRNAs able to simultaneously silence the expression of viral proteins and cellular proteins involved in pathways leading to the inflammatory/antiviral response against HRSV. Alignment of the mRNA sequences of some of these cellular proteins with the HRSV genome (positive polarity) showed that there were short segments highly conserved between them that could be targeted by bispecific siRNAs. Based on this, two siRNAs were obtained that silenced TRIM25 and the HRSV F while also silencing RIG-I and HRSV N.

Small interfering RNAs have several advantages as potential treatments against diverse pathogens. They are cheap and easy to produce, they can be lyophilized to increase their stability, and they are highly specific. An additional advantage in the case of respiratory viruses is that they can be administered locally to the respiratory tract. Furthermore, they can be easily delivered as combinations of siRNAs targeting distinct regions in the same or different viral genes. This will improve the efficacy of the treatment while expanding the spectrum of susceptible genotypes and limiting the emergence of treatment-resistant variants [42]. Therefore, it is not surprising that the number of published clinical studies using siRNAs has increased remarkably in recent years [19]. Despite all of this, off-target effects of siRNAs still remain an important problem to deal with. We performed a cytotoxic assay that ruled out any mayor effect of the siRNAs tested on the cell viability. In addition, these siRNAs do not seem to induce a significant interferon response leading to a general translation arrest since: (1) in the case of cells transfected with Control, RIG-I and TRIM25 siRNAs, viral protein levels increased from 24 hpi. to 48 hpi (Figure 4); (2) HRSV-F and TRIM25/HRSV-F siRNAs inhibited only the expression of the F protein, but not the accumulation of the other viral proteins (Figure 4); and (3) the antiviral activity of HRSV-N (ALN-RSV01) has not been shown to be mediated by interferon [27]. However, it is difficult to completely rule out any off-target effect of siRNAs on host genes, since the siRNA guide strand may silence unintended mRNAs by a mechanism similar to micro RNA silencing, in which only partial sequence complementarity, mainly mediated by the seed sequence (nucleotides 2–8), is required [39].

Both virus replication and an inadequate immune response seem to be responsible for the pathology caused by HRSV [43]. Thus, targeting these two elements of HRSV infection could improve virus clearance and ameliorate disease symptoms. Khaitov et al. have reported that the combined administration of siRNAs targeting HRSV and IL4 reduced inflammation in a mouse model of asthma exacerbation, in which mice were sensitized with ovalbumin before HRSV infection [44]. The host immunity against HRSV is initiated in the infected pulmonary epithelial cells, the primary targets of HRSV infection. These cells secrete pro-inflammatory cytokines and chemokines that promote tissue inflammation and contribute to the induction of the subsequent adaptive response. Therefore, in the same lung epithelial cell, both HRSV replication and the early innate immune response can be targeted at the same time. We have used a lung epithelial cell line to analyze the simultaneous inhibition of HRSV growth and the cellular immune response with bispecific siRNAs. Although bispecific siRNAs and the combination of siRNAs against both the virus and the innate immune genes reduced virus production and the inflammatory/antiviral response in infected cells, it was found that monospecific siRNAs silencing viral genes alone reduced the intracellular innate response. However, our results showed that the quantitative impact of these siRNAs on the immune response differed considerably. This may be related to the role of the viral protein in the life cycle of the virus, as pointed by Bitko and Barik [21]. Thus, the inhibition of N expression reduced viral replication and, therefore, the amount of total viral RNAs. Since these RNAs act as substrates for PRRs [45], N silencing resulted in a drastic reduction in the cellular innate immune response. By contrast, silencing HRSV F, which is not involved in HRSV replication/transcription, did not result in a generalized viral RNA decrease, and intracellular signaling mediated by PRRs continued, to some extent, in these siRNA-transfected cells. This strongly indicates that targeting distinct HRSV genes would lead to different inflammatory/antiviral response outcomes without compromising viral titer reduction. Our results are in line with those of a recent report in which the interferon response induced by HRSV in epithelial cells was modulated by target-specific antiviral treatment with small-molecule inhibitors [46]. An HRSV polymerase (L) inhibitor leading to the accumulation of abortive viral RNAs induced the upregulation of interferon-stimulated genes, while another L inhibitor leading to overall suppression of HRSV RNA synthesis resulted in the attenuation of the RIG-I-like pathway [46].

The immune response against HRSV is complex and several viral and host factors have been related to HRSV immunopathology [43]. Protective immunity includes virus-neutralizing antibodies, mostly against the F protein [2]. However, it is not clear if a potent early innate immune response is beneficial or detrimental to prevent from severe HRSV infections [47]. Furthermore, it is probable that some aspects of that response are beneficial and others are not, and this may depend on the infected person. Therefore, although we have focused on reducing an early innate response against the HRSV, it is possible that, in some circumstances, a strong response is required. In this case, it would be desirable that the silencing of the HRSV genes does not significantly reduce this response. In addition, high expression of the viral glycoproteins (F and G), which are targets for neutralizing antibodies, should be maintained. As shown in the present report, silencing HRSV N drastically reduced the innate immune response, as well as the expression of F and G. On the contrary, silencing F had a moderate effect on the innate immune response but drastically reduced the expression of the protein F, which is the main target for neutralizing antibodies. Previous studies using siRNAs against HRSV have focused on proteins involved in replication/transcription (N, P, and M2-2) [26,27,30,32,34] or the fusion F protein [30,33]. In all cases, a substantial decrease in virus titers has been reported. However, in most reports, the early innate immune response has not been assessed, which precludes a direct comparison with our data. In any case, it would be interesting to silence other HRSV genes not involved in virus replication/transcription or the induction of neutralizing antibodies, such as the matrix (M). M silencing would allow both the accumulation of enough RNA and viral glycoproteins for the development of the innate immune response and the induction of neutralizing antibodies. An additional advantage of the HRSV M protein is that it is one of the most conserved proteins of the virus [48], so it would be easier to design an siRNA effective against most HRSV isolates. Another approach is targeting non-structural HRSV genes that antagonize the host IFN response. For example, it has been reported that an siRNA against the HRSV NS1 reduced virus replication in A549 cells and mice, while upregulating the expression of IFN-β [25]. However, the reduction in HRSV production may have been due to the upregulation of the innate immune response rather than due to the direct silencing of viral RNA [25].

As mentioned before, siRNAs have several advantages, including that they can be easily designed to target almost any viral or cellular gene. This offers a plethora of alternatives to treat viral or other pathogen infections while fine-tuning the associated immune/antiviral response. Since intracellular immune pathways are tightly regulated by positive and negative regulators, different combinations of siRNAs against viral and cellular genes can be envisaged to inhibit virus growth and to reduce or potentiate the inflammatory/antiviral response as needed. However, this versatility will likely require the use of combination of siRNAs against viral and cellular siRNAs rather than bispecific siRNAs since the latter are restricted by the existence of sequence similarities between the viral and cellular genes of interest.

Lastly, as shown in the present study and in other studies [25,27,32], siRNAs against HRSV are highly effective when administered before infection, but they also work when delivered after infection, which suggests that they could be useful both in prophylactic and therapeutic treatments.

## 5. Conclusions

In conclusion, although several studies have reported that different siRNAs against HRSV reduced virus growth, our results show that the effect of those siRNAs should be assessed not only by taking into account virus replication but also by taking into account their effects on the immune response. In addition, different combinations of viral and cellular gene silencing may provide us with specific tools to control the undesired aspect of the immune response against HRSV while potentiating the protective ones. This suggests new avenues to explore in the design of novel antiviral treatments based on siRNAs, which may contribute to a better management of HRSV infections.

## Figures and Tables

**Table 1:** Nucleotide sequences of the siRNAs.

Name *^a^*	Sense Sequence (5′-3′) *^b^*	Antisense Sequence (5′-3′) *^b^*
TRIM25	CAACAAGAAUACACGGAAAtt	UUUCCGUGUAUUCUUGUUGta
HRSV-F	GCAAAGUGUUAGACCUCAAtt	UUGAGGUCUAACACUUUGCtg
TRIM25/HRSV-F	CCAUAGACCUCAAAAACGAtt	UCGUUUUUGAGGUCUAUGGtg
RIG-I	CAAGAAGAGUACCACUUAAtt	UUAAGUGGUACUCUUCUUGta
HRSV-N (ALN-RSV01)	GGCUCUUAGCAAAGUCAAGtt	CUUGACUUUGCUAAGAGCCtt
RIG-I/HRSV-N	GGAAAUGGAGCAAGUUGUUtt	AACAACUUGCUCCAUUUCCtc

*^a^* TRIM25: tripartite motif-containing protein 25. RIG-I: retinoic acid-inducible gene-I. HRSV-F: human respiratory syncytial virus fusion protein. HRSV-N: human respiratory syncytial virus nucleoprotein. *^b^* Dinucleotide overhangs are in lowercase.

## Supplementary Materials

The following are available online at https://www.mdpi.com/2218-273X/9/5/165/s1, Supplemental Figure S1: (a) Relative levels of TRIM25 or HRSV-F (upper panel) and RIG-I or HRSV-N (lower panel) in cells transfected with the siRNAs shown in parentheses. Levels of mRNAs were quantified by qRT-PCR 48 h post-transfection and represented as folds over mRNAs expressed in cells transfected with a control siRNA. Data represent the mean and standard deviation from three independent experiments. (b) Relative levels of TRIM25 or RIG-I in cells silenced with the siRNAs shown in parenthesis. Levels of mRNAs were quantified by qRT-PCR 24 h post-transfection (before infection) and represented as folds over mRNAs expressed in cells transfected with a control siRNA. Data represent the mean and standard deviation from triplicate transfections. Supplemental Figure S2: Coding Sequences (CDS) of TRIM25 and HRSV-F showing the target sequence of TRIM25 siRNA (green), HRSV-F siRNA (blue), and TRIM25/HRSV-F siRNA (red). Mismatches between target genes and the siRNA are in lowercase. Supplemental Figure S3: Coding Sequences (CDS) of RIG-I and HRSV-N showing the target sequence of RIG-I siRNA (green), HRSV-N siRNA (blue), and RIG-I/HRSV-N siRNA (red). Mismatches between target genes and the siRNA are in lowercase. Supplemental Figure S4: Percentage of viable cells (survival rate, SR) transfected with the different siRNAs at 0 and 72 h post-transfection. An MTT assay determined cell viability. Data represent the mean and standard deviation from quintuplicate transfections. A dilution of 1:2000 (from a 30% stock) of H_2_O_2_ was used as a positive control of cell damage.
