# Supplementary material for: siRNA-Mediated Simultaneous Regulation of the Cellular Innate Immune Response and Human Respiratory Syncytial Virus Replication
Source: Biomolecules. 2019 Apr 28;9(5):165. doi: 10.3390/biom9050165 (PMC6572644; doi:10.3390/biom9050165)
Supplement: Supplementary file 1 [file biomolecules-09-00165-s001.pdf]

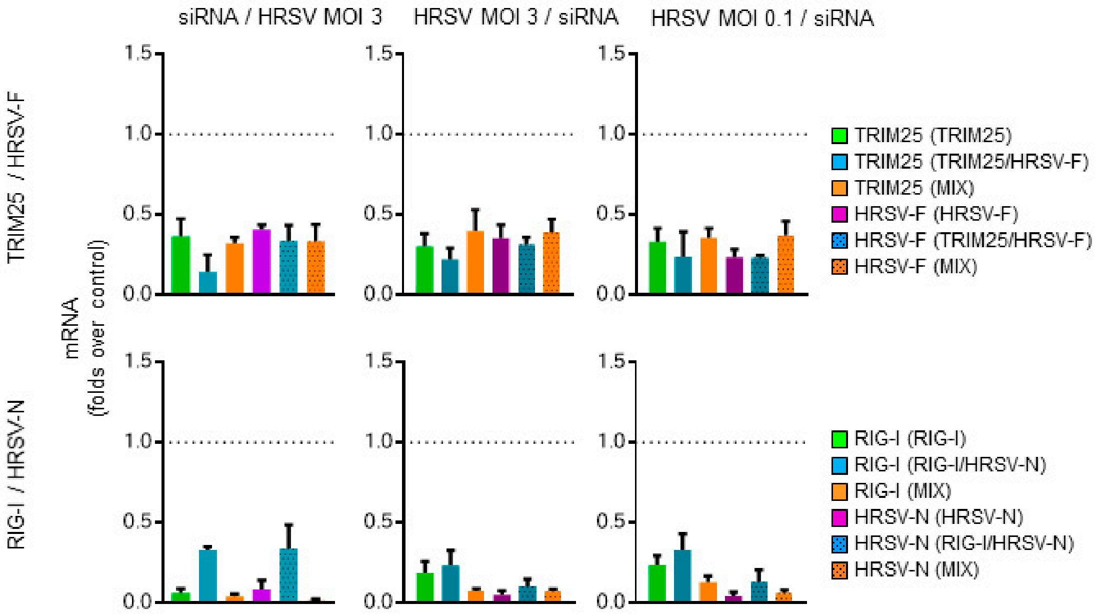

Supplemental Figure 1a. Relative levels of TRIM25 or HRSV-F (upper panel) and RIG-I or HRSV-N (lower panel) in cells silenced with the siRNAs shown in parenthesis. Levels of mRNAs were quantified by qRT-PCR at 48 h post-transfection and represented as folds over mRNAs expressed in cells transfected with a control siRNA. Data represent the mean and standard deviation from three independent experiments.

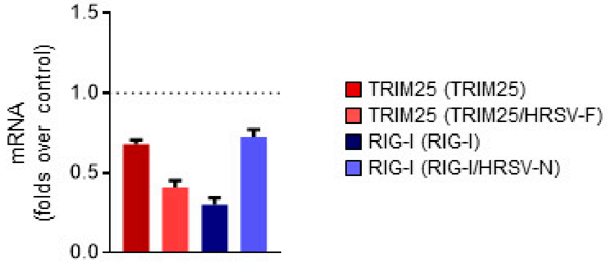

Supplemental Figure 1b. Relative levels of TRIM25 or RIG-I in cells silenced with the siRNAs shown in parenthesis. Levels of mRNAs were quantified by qRT-PCR at 24 h post-transfection (before infection) and represented as folds over mRNAs expressed in cells transfected with a control siRNA. Data represent the mean and standard deviation from triplicate transfections.

CDS TRIM25

ATGGCAGAGCTGTGCCCCCTGGCCGAGGAGCTGTCGTGCTCCATCTGCCTGGAGCCCTTCAAGGAGCCGGTCACCACTCCGTGCGGCCACAACTTCTGCGGGTC
GTGCCTGAATGAGACGTGGGCAGTCCAGGGCTCGCCATACCTGTGCCCGCAGTGCCGCGCCGTCTACCAGGCGCGACCGCAGCTGCACAAGAACACGGTGCT
GTGCAACGTGGTGGAGCAGTTCCTGCAGGCCGACCTGGCCCGGGAGCCACCCGCCGACGTCTGGACGCCGCCCGCCCGCGCCTCTGCACCCAGCCCGAATGCC
CAGGTGGCCTGCGACCACTGCCTGAAGGAGGCCGCCGTGAAGACGTGCTTGGTGTGCATGGCCTCCTTCTGTCAGGAGCACCTGCAGCCGCACTTCGACAGCC
CCGCCTTCCAGGACCACCCGCTGCAGCCGCCCGTTCGCGACCTGTTGCGCCGCAAATGTTCCCAGCACAATCGGCTGCGGGAATTTTTCTGCCCCGAGCACAGC
GAGTGCATCTGCCACATCTGCCTGGTGGAGCATAAGACCTGCTCTCCCGCGTCCCTGAGCCAGGCCAGCGCCGACCTGGAGGCCACCCTGAGGCACAAACTAA
CTGTCATGTACAGTCAGATCAACGGGGCGTCGAGAGCACTGGATGATGTGAGAAACAGGCAGCAGGATGTGCGGATGACTGCAAACAGAAAGGTGGAGCAG
CTA**CAACAAGAATACACGGAAA**TGAAGGCTCTCTTGGACGCCTCAGAGACCACCTCGACAAGGAAGATAAAGGAAGAGGAGAAGAGGGTCAACAGCAAGTT
TGACACCATTTATCAGATTCTCCTCAAGAAGAAGAGTGAGATCCAGACCTTGAAGGAGGAGATTGAACAGAGCCTGACCAAGAGGGATGAGTTCGAGTTTCTG
GAGAAAGCATCAAAACTGCGAGGAATCTCAACAAAGCCAGTCTACATCCCCGAGGTGGAACTGAACCACAAGCTGATAAAAGGCATCCACCAGAGCA**CCATA**
GACCTCAAAAACGAGCTGAAGCAGTGCATCGGGCGGCTCCAGGAGCCCACCCCCAGTTCAGGTGACCCTGGAGAGCATGACCCAGCGTCCACACACAAATCC
ACACGCCCTGTGAAGAAGGTCTCCAAAGAGGAAAAGAAATCCAAGAAACCTCCCCCTGTCCCTGCCTTACCCAGCAAGCTTCCCACGTTTGGAGCCCCGGAACA
GTTAGTGGATTTAAAACAAGCTGGCTTGGAGGCTGCAGCCAAAGCCACCAGCTCACATCCGAACTCAACATCTCTCAAGGCCAAGGTGCTGGAGACCTTCCTG
GCCAAGTCCAGACCTGAGCTCCTGGAGTATTACATTAAAGTCATCCTGGACTACAACACCGCCCACAACAAAGTGGCTCTGTCAGAGTGCTATACAGTAGCTTC
TGTGGCTGAGATGCCTCAGAACTACCGGCCGCATCCCCAGAGGTTCACATACTGCTCTCAGGTGCTGGGCCTGCACTGCTACAAGAAGGGGATCCACTACTGG
GAGGTGGAGCTGCAGAAGAACAACTTCTGTGGGGTAGGCATCTGCTACGGAAGCATGAACCGGCAGGGCCCAGAAAGCAGGCTCGGCCGCAACAGCGCCTC
CTGGTGCGTGGAGTGGTTCAACACCAAGATCTCTGCCTGGCACAATAACGTGGAGAAAACCCTGCCCTCCACCAAGGCCACGCGGGTGGGCGTGCTTCTCAAC
TGTGACCACGGCTTTGTCATCTTCTTCGCTGTTGCCGACAAGGTCCACCTGATGTATAAGTTCAGGGTGGACTTTACTGAGGCTTTGTACCCGGCTTTCTGGGTA
TTTTCTGCTGGTGCCACACTCTCCATCTGCTCCCCCAAGTAGGCAGGCT

CDS HRSV-F

ATGGAGTTGCCAATCCTCAAAGCAAATGCAATTACCACAATCCTCGCTGCAGTCACATTTTGCTTTGCTTCTAGTCAAAACATCACTGAAGAATTTTATCA
ATCAACATGCAGTGCAGTTAGCAAAGGCTATCTTAGTGCTCTAAGGACTGGTTGGTATACTAGTGTTATAACTATAGAATTAAGTAATATCAAGGAAAA
TAAGTGTAATGGAACAGATGCCAAGGTAAAACTGATAAAACAAGAATTAGATAAATATAAAAATGCTGTAACAGAATTGCAGTTGCTCATGCAAAGCA
CACCAGCAGCAAACAATCGAGCCAGAAGAGAACTACCAAGGTTTATGAATTATACACTCAACAATACCAAAAAAACCAATGTAACATTAAGCAAGAAA
AGGAAAAGAAGATTTCTTGGTTTTTTGTTAGGTGTTGGATCTGCAATCGCCAGTGGCACTGCTGTATCTAAGGTCCTGCACTTAGAAGGAGAAGTGAAC
AAGATCAAAAGTGCTCTACTATCCACAAACAAGGCCGTAGTCAGCTTATCAAATGGAGTTAGTGTCTTAACCA**GCAAAGTGTTAGACCTCAA**AAACTAT
ATAGATAAACAATTGTTACCTATTGTGAATAAGCAAAGCTGCAGAATATCAAATATAGAAACTGTGATAGAGTTCCAACAAAAGAACAACAGACTACT
AGAGATTACCAGGGAATTTAGTGTTAATGCAGGTGTAACTACACCTGTAAGCACTTACATGTTAACTAATAGTGAATTATTGTCATTAATCAATGATATG
CCTATAACAAATGATCAGAAAAAGTTAATGTCCAACAATGTTCAAATAGTTAGACAGCAAAGTTACTCTATCATGTCCATAATAAAAGAGGAAGTCTTA
GCATATGTAGTACAATTACCACTATATGGTGTGATAGATACACCTTGTTGGAAATTACACACATCCCCTCTATGTACAACCAACACAAAAGAAGGGTCA
AACATCTGTTTAACAAGAACTGACAGAGGATGGTACTGTGACAATGCAGGATCAGTATCTTTCTTCCCACAAGCTGAAACATGTAAAGTTCAATCGAAT
CGAGTATTTTGTGACACAATGAACAGTTTAACATTACCAAGTGAAGTAAATCTCTGCAATGTTGACATATTCAATCCCAAATATGATTGTAAAATTATGA
CTTCAAAAAACAGATGTAAGCAGCTCCGTTATCACATCTCTAGGAGCCATTGTGTCATGCTATGGCAAAACTAAATGTACAGCATCCAATAAAAATCGTG
GAATCATAAAGACATTTTCTAACGGGTGTGATTATGTATCAAATAAAGGGGTGGACACTGTGTCTGTAGGTAACACATTATATTATGTAAATAAGCAAG
AAGGCAAAAGTCTCTATGTAAAAGGTGAACCAATAATAAATTTCTATGACCCATTAGTATTCCCCTCTGATGAATTTGATGCATCAATATCTCAAGTCAA
TGAGAAGATTAACCAGAGTTTAGCATTTATTCGTAAATCCGATGAATTATTACATCATGTAAATGCTGGTAAATCAACCACAAATATCATGATAACTACT
ATAATTATAGTGATTATAGTAATATTGTTATCATTAATTGCTGTTGGACTGCTCCTATACTGTAAGGCCAGAAGCACACCAGTCACACTAAGCAAGGATC
AACTGAGTGGTATAAATAATATTGCATTTAGTAACTGAAT

CDS HRSV-F

ATGGAGTTGCCAATCCTCAAAGCAAATGCAATTACCACAATCCTCGCTGCAGTCACATTTTGCTTTGCTTCTAGTCAAAACATCACTGAAGAATTTTATCA
ATCAACATGCAGTGCAGTTAGCAAAGGCTATCTTAGTGCTCTAAGGACTGGTTGGTATACTAGTGTTATAACTATAGAATTAAGTAATATCAAGGAAAA
TAAGTGTAATGGAACAGATGCCAAGGTAAAACTGATAAAACAAGAATTAGATAAATATAAAAATGCTGTAACAGAATTGCAGTTGCTCATGCAAAGCA
CACCAGCAGCAAACAATCGAGCCAGAAGAGAACTACCAAGGTTTATGAATTATACACTCAACAATACCAAAAAAACCAATGTAACATTAAGCAAGAAA
AGGAAAAGAAGATTTCTTGGTTTTTTGTTAGGTGTTGGATCTGCAATCGCCAGTGGCACTGCTGTATCTAAGGTCCTGCACTTAGAAGGAGAAGTGAAC
AAGATCAAAAGTGCTCTACTATCCACAAACAAGGCCGTAGTCAGCTTATCAAATGGAGTTAGTGTCTTAACCAGCAAAG**tgtTAGACCTCAAAAACtA**TA
TAGATAAACAATTGTTACCTATTGTGAATAAGCAAAGCTGCAGAATATCAAATATAGAAACTGTGATAGAGTTCCAACAAAAGAACAACAGACTACTA
GAGATTACCAGGGAATTTAGTGTTAATGCAGGTGTAACTACACCTGTAAGCACTTACATGTTAACTAATAGTGAATTATTGTCATTAATCAATGATATGC
CTATAACAAATGATCAGAAAAAGTTAATGTCCAACAATGTTCAAATAGTTAGACAGCAAAGTTACTCTATCATGTCCATAATAAAAGAGGAAGTCTTAG
CATATGTAGTACAATTACCACTATATGGTGTGATAGATACACCTTGTTGGAAATTACACACATCCCCTCTATGTACAACCAACACAAAAGAAGGGTCAA
ACATCTGTTTAACAAGAACTGACAGAGGATGGTACTGTGACAATGCAGGATCAGTATCTTTCTTCCCACAAGCTGAAACATGTAAAGTTCAATCGAATC
GAGTATTTTGTGACACAATGAACAGTTTAACATTACCAAGTGAAGTAAATCTCTGCAATGTTGACATATTCAATCCCAAATATGATTGTAAAATTATGAC
TTCAAAAAACAGATGTAAGCAGCTCCGTTATCACATCTCTAGGAGCCATTGTGTCATGCTATGGCAAAACTAAATGTACAGCATCCAATAAAAATCGTGG
AATCATAAAGACATTTTCTAACGGGTGTGATTATGTATCAAATAAAGGGGTGGACACTGTGTCTGTAGGTAACACATTATATTATGTAAATAAGCAAGA
AGGCAAAAGTCTCTATGTAAAAGGTGAACCAATAATAAATTTCTATGACCCATTAGTATTCCCCTCTGATGAATTTGATGCATCAATATCTCAAGTCAAT
GAGAAGATTAACCAGAGTTTAGCATTTATTCGTAAATCCGATGAATTATTACATCATGTAAATGCTGGTAAATCAACCACAAATATCATGATAACTACTA
TAATTATAGTGATTATAGTAATATTGTTATCATTAATTGCTGTTGGACTGCTCCTATACTGTAAGGCCAGAAGCACACCAGTCACACTAAGCAAGGATCA
ACTGAGTGGTATAAATAATATTGCATTTAGTAACTGAAT

Supplemental Figure 2. Coding Sequences (CDS) of *TRIM25* and *HRSV-F* showing the target sequence of TRIM25 (green) siRNA, HRSV-F siRNA (blue) and TRIM25 / HRSV-F siRNA (red). Mismatches between target genes and the siRNA are in lowercase.

CDS RIG-I

ATGACCACCGAGCAGCGACGCAGCCTGCAAGCCTTCCAGGATTATATCCGGAAGACCCTGGACCCTACCTACATCCTGAGCTACATGGCCCCCTGGTTTA
GGGAGGAAGAGGTGCAGTATATTCAGGCTGAGAAAAACAACAAGGGCCCAATGGAGGCTGCCACACTTTTTCTCAAGTTCCTGTTGGAGCTCCAGGAGG
AAGGCTGGTTCCGTGGCTTTTTGGATGCCCTAGACCATGCAGGTTATTCTGGACTTTATGAAGCCATTGAAAGTTGGGATTTCAAAAAAATTGAAAAGTT
GGAGGAGTATAGATTACTTTTAAAACGTTTACAACCAGAATTTAAAACCAGAATTATCCCAACCGATATCATTTCTGATCTGTCTGAATGTTTAATTAATCA
GGAATGTGAAGAAATTCTACAGATTTGCTCTACTAAGGGGATGATGGCAGGTGCAGAGAAATTGGTGGAATGCCTTCTCAGATCAGACAAGGAAAACTG
GCCCAAAACTTTGAAACTTGCTTTGGAGAAAGAAAGGAACAAGTTCAGTGAACTGTGGATTGTAGAGAAAGGTATAAAAGATGTTGAAACAGAAGATCT
TGAGGATAAGATGGAAACTTCTGACATACAGATTTTCTACCAAGAAGATCCAGAATGCCAGAATCTTAGTGAGAATTCATGTCCACCTTCAGAAGTGTCT
GATACAAACTTGTACAGCCCATTTAAACCAAGAAATTACCAATTAGAGCTTGCTTTGCCTGCTATGAAAGGAAAAAACACAATAATATGTGCTCCTACAGG
TTGTGGAAAAACCTTTGTTTCACTGCTTATATGTGAACATCATCTTAAAAAATTCCCACAAGGACAAAAGGGGAAAGTTGTCTTTTTTGCGAATCAGATCC
CAGTGTATGAACAGCAGAAATCTGTATTCTCAAAATACTTTGAAAGACATGGGTATAGAGTTACAGGCATTTCTGGAGCAACAGCTGAGAATGTCCCAGT
GGAACAGATTGTTGAGAACAATGACATCATCATTTTAACTCCACAGATTCTTGTGAACAACCTTAAAAAGGGAACGATTCCATCACTATCCATCTTTACTTT
GATGATATTTGATGAATGCCACAACACTAGTAAACAACACCCGTACAATATGATCATGTTTAATTATCTAGATCAGAAACTTGGAGGATCTTCAGGCCCAC
TGCCCCAGGTCATTGGGCTGACTGCCTCGGTTGGTGTTGGGGATGCCAAAAACACAGATGAAGCCTTGGATTATATCTGCAAGCTGTGTGCTTCTCTTGAT
GCGTCAGTGATAGCAACAGTCAAACACAATCTGGA**GGAAcTGGAGCAAGTTGTT**TATAAGCCCCAGAAGTTTTTCAGGAAAGTGGAATCACGGATTAGC
GACAAATTTAAATACATCATAGCTCAGCTGATGAGGGACACAGAGAGTCTGGCAAAGAGAATCTGCAAAGACCTCGAAAACTTATCTCAAATTCAAAATA
GGGAATTTGGAACACAGAAATATGAACAATGGATTGTTACAGTTCAGAAAGCATGCATGGTGTTCCAGATGCCAGACAAAGATGAAGAGAGCAGGATTT
GTAAAGCCCTGTTTTTATACACTTCACATTTGCGGAAATATAATGATGCCCTCATTATCAGTGAGCATGCACGAATGAAAGATGCTCTGGATTACTTGAAA
GACTTCTTCAGCAATGTCCGAGCAGCAGGATTCGATGAGATTGAGCAAGATCTTACTCAGAGATTTGAAGAAAAGCTGCAGGAACTAGAAAGTGTTTCCA
GGGATCCCAGCAATGAGAATCCTAAACTTGAAGACCTCTGCTTCATCTTA**CAAGAAGAGTACCACTTAA**ACCCAGAGACAATAACAATTCTCTTTGTGAA
AACCAGAGCACTTGTGGACGCTTTAAAAAATTGGATTGAAGGAAATCCTAAACTCAGTTTTCTAAAACCTGGCATATTGACTGGACGTGGCAAAACAAAT
CAGAACACAGGAATGACCCTCCCGGCACAGAAGTGTATATTGGATGCATTCAAAGCCAGTGGAGATCACAATATTCTGATTGCCACCTCAGTTGCTGATG
AAGGCATTGACATTGCACAGTGCAATCTTGTCATCCTTTATGAGTATGTGGGCAATGTCATCAAAATGATCCAAACCAGAGGCAGAGGAAGAGCAAGAG
GTAGCAAGTGCTTCCTTCTGACTAGTAATGCTGGTGTAATTGAAAAAGAACAAATAAACATGTACAAAGAAAAAATGATGAATGACTCTATTTTACGCCTT
CAGACATGGGACGAAGCAGTATTTAGGGAAAAGATTCTGCATATACAGACTCATGAAAAATTCATCAGAGATAGTCAAGAAAAACCAAAACCTGTACCT
GATAAGGAAAATAAAAAACTGCTCTGCAGAAAGTGCAAAGCCTTGGCATGTTACACAGCTGACGTAAGAGTGATAGAGGAATGCCATTACACTGTGCTT
GGAGATGCTTTTAAGGAATGCTTTGTGAGTAGACCACATCCCAAGCCAAAGCAGTTTTCAAGTTTTGAAAAAAGAGCAAAGATATTCTGTGCCCGACAGA
ACTGCAGCCATGACTGGGGAATCCATGTGAAGTACAAGACATTTGAGATTCCAGTTATAAAAATTGAAAGTTTTGTGGTGGAGGATATTGCAACTGGAGT
TCAGACACTGTACTCGAAGTGGAAGGACTTTCATTTTGAGAAGATACCATTTGATCCAGCAGAAATGTCCAAATGA

CDS RSV-N

AT**GGCTCTTAGCAAAGTCAAG**TTGAATGATACACTCAACAAAGATCAACTTCTGTCATCCAGCAAATACGCCATCCAACGGAGCACAGGAGATAGTATTG
ATACTCCTAATTATGATGTGCAGAAACACATCAATAAGTTATGTGGCATGTTATTAATCACAGAAGATGCTAATCATAAATTCACTGGGTTAATAGGTATG
TTATATGCGATGTCTAGGTTAGGAAGAGAAGACACCATAAAAATACTCAGAGATGCGGGATATCATGTAAAAGCAAATGGAGTAGATGTAACAACACAT
CGTCAAGACATTAATGGAAAAGAAATGAAATTTGAAGTGTTAACATTGGCAAGCTTAACAACTGAAATTCAAATCAACATTGAGATAGAATCTAGAAAAT
CCTACAAAAAAATGCTAAAAGAAATGGGAGAGGTAGCTCCAGAATACAGGCATGACTCTCCTGATTGTGGGATGATAATATTATGTATAGCAGCATTAGT
AATAACTAAATTAGCAGCAGGGGACAGATCTGGTCTTACAGCCGTGATTAGGAGAGCTAATAATGTCCTAAAAAATGAAATGAAACGTTACAAAGGCTT
ACTACCCAAGGACATAGCCAACAGCTTCTATGAAGTGTTTGAAAAACATCCCCACTTTATAGATGTTTTTGTTCATTTTGGTATAGCACAATCTTCTACCAG
AGGTGGCAGTAGAGTTGAAGGGATTTTTGCAGGATTGTTTATGAATGCCTATGGTGCAGGGCAAGTGATGTTACGGTGGGGAGTCTTAGCAAAATCAGT
TAAAAATATTATGTTAGGACATGCTAGTGTGCAAGC**aGAAATGGAaCAAGTTGTT**GAGGTTTATGAATATGCCCAAAAATTGGGTGGTGAAGCAGGATT
CTACCATATATTGAACAACCCAAAAGCATCATTATTATCTTTGACTCAATTTCCTCACTTCTCCAGTGTAGTATTAGGCAATGCTGCTGGCCTAGGCATAAT
GGGAGAGTACAGAGGTACACCGAGGAATCAAGATCTATATGATGCAGCAAAGGCATATGCTGAACAACTCAAAGAAAATGGTGTGATTAACTACAGTGT
ACTAGACTTGACAGCAGAAGAACTAGAGGCTATCAAACATCAGCTTAATCCAAAAGATAATGATGTAGAGCTTTGA

Supplemental Figure 3. Coding Sequences (CDS) of *RIG-I* and *HRSV-N* showing the target sequence of RIG-I (green) siRNA, HRSV-N siRNA (blue) and RIG-I / HRSV-N siRNA (red). Mismatches between target genes and the siRNA are in lowercase.

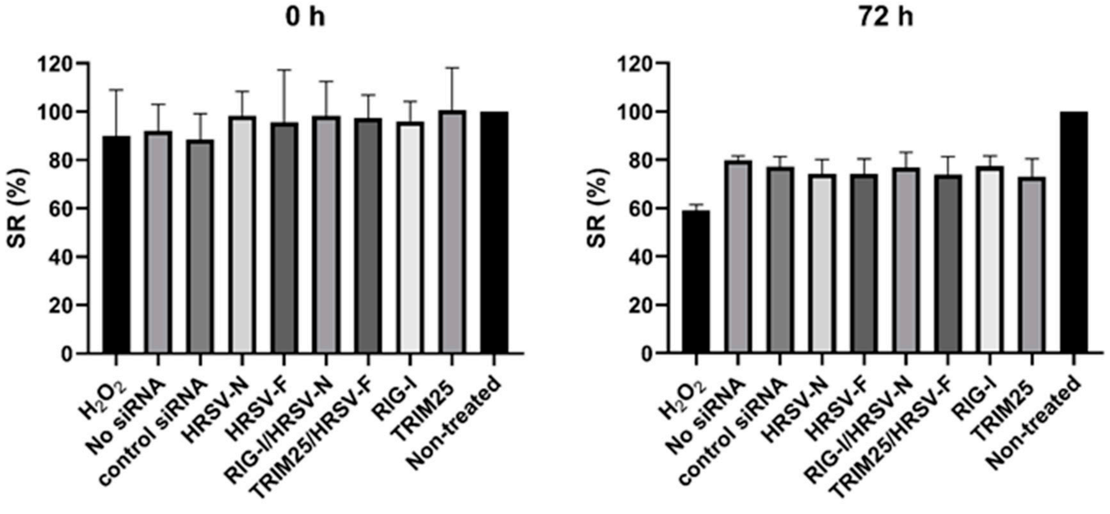

Supplemental Figure 4. Percentage of viable cells (survival rate, SR) transfected with the different siRNAs at 0 and 72 h post-transfection. Cell viability was determined by a MTT assay. Data represent the mean and standard deviation from quintuplicate transfections. A dilution of 1:2000 (from a 30% stock) of H_2O_2 was used as a positive control of cell damage.
